# Nanoparticle-Mediated Delivery Systems in Photodynamic Therapy of Colorectal Cancer

**DOI:** 10.3390/ijms222212405

**Published:** 2021-11-17

**Authors:** Nokuphila Winifred Nompumelelo Simelane, Heidi Abrahamse

**Affiliations:** Laser Research Centre, Faculty of Health Sciences, University of Johannesburg, P.O. Box 17011, Doornfontein 2028, South Africa; 201904217@student.uj.ac.za

**Keywords:** colorectal cancer, photodynamic therapy, photosensitizers, nanotechnology, 3D models

## Abstract

Colorectal cancer (CRC) involving a malignant tumour remains one of the greatest contributing causes of fatal mortality and has become the third globally ranked malignancy in terms of cancer-associated deaths. Conventional CRC treatment approaches such as surgery, radiation, and chemotherapy are the most utilized approaches to treat this disease. However, they are limited by low selectivity and systemic toxicity, so they cannot completely eradicate this disease. Photodynamic therapy (PDT) is an emerging therapeutic modality that exerts selective cytotoxicity to cancerous cells through the activation of photosensitizers (PSs) under light irradiation to produce cytotoxic reactive oxygen species (ROS), which then cause cancer cell death. Cumulative research findings have highlighted the significant role of traditional PDT in CRC treatment; however, the therapeutic efficacy of the classical PDT strategy is restricted due to skin photosensitivity, poor cancerous tissue specificity, and limited penetration of light. The application of nanoparticles in PDT can mitigate some of these shortcomings and enhance the targeting ability of PS in order to effectively use PDT against CRC as well as to reduce systemic side effects. Although 2D culture models are widely used in cancer research, they have some limitations. Therefore, 3D models in CRC PDT, particularly multicellular tumour spheroids (MCTS), have attracted researchers. This review summarizes several photosensitizers that are currently used in CRC PDT and gives an overview of recent advances in nanoparticle application for enhanced CRC PDT. In addition, the progress of 3D-model applications in CRC PDT is discussed.

## 1. Introduction

Cancer has become the second largest lethal disease that causes fatal mortality, worldwide [1]. As an extremely heterogeneous disease, cancer is characterized by uncontrolled growth proliferation within different tissues and the tendency to metastasize, resulting in tumour-related death [2]. Colorectal cancer (CRC) has become the fourth most commonly diagnosed and the third most fatal malignancy, with a steady rise of more than a million new cases excepted each year [3]. Despite promising advancements in the standard treatment approaches, a higher mortality rate is still associated with CRC [3].

**Figure 1:**
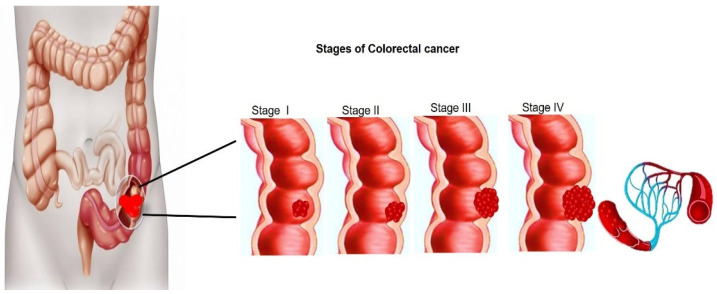
Different stages of colorectal cancer using the American Joint Committee (AJCC) on Cancer based on tumour, nodes, metastasis (TNM) classification.

Generally, more than 90% of CRCs are adenocarcinomas that originate from the mucosal epithelial cells of the surrounding colon surface [4]. The cancer arises when certain cells of the mucosal epithelium transform into malignant unregulated polyps after acquiring a series of genetic or epigenetic mutations [3]. Although approximately 10% of polyps may progress to become cancerous, if they are not eliminated, these malignant cells may develop the ability to spread into the walls of the colon and potentially invade beyond the colorectal wall, thereby promoting metastases to distant organs [3]. CRC can be classified into four stages based on the guidelines from the American Joint Committee on Cancer, beginning with stage 0, which is the earliest and least advanced stage of CRCs (localized cancer); CRC then progresses through the early stages (I to II-C) to the most advanced stages (III-A to IV-B) (Figure 1) [5]. Several genetic and environmental factors that can exacerbate CRC development include active smoking, heavy alcohol consumption, poor diet, advancing age, obesity, and environmental toxicity [3].

CRC survival is highly dependent on the stage of the malignancy at diagnosis, and when the cancer is at a localized stage, it is associated with an improved 5-year survival rate of up to 90% [6]. However, advanced stages of CRC often lack the chance for an efficient therapeutic intervention [7]. Early diagnosis of CRC is therefore of great importance, even in an asymptomatic or benign stage, to yield better outcomes [7]. Currently, traditional modalities for CRC early diagnosis include colonoscopy, sigmoidoscopy, faecal occult blood test (FOBT), and faecal immunochemical tests (FITs) [7]. However, they are associated with several drawbacks such as bowel perforation, invasiveness, and unpleasantness [7].

Within the conventional therapeutic modalities for CRC, surgery, chemotherapy, and radiotherapy have been established as the widely used treatments [5]. Surgery is a standard option for the treatment of earlier stages of CRC development, potentially preventing recurrence as well as increasing the CRC survival rate [5]. However, it still presents some unfavourable drawbacks such as colon haemorrhages in poorly located cancerous tissues [5]. Furthermore, the treatment efficacy of traditional CRC therapies, such as chemotherapy and radiotherapy, are associated with various complications, such as severe adverse systemic effects, toxicity, non-specific cancer-targeting abilities, and multidrug resistance [8]. In addition to the traditional treatment modalities, cellular therapy, gene therapy, immunotherapy, and targeted therapy have exhibited outstanding potential in CRC treatment [8]. These strategies seek to boost the immune system’s ability to recognize cancer cells and fortify its response [8]. The immune system can then initiate various mechanisms that can improve the accumulation of antitumour agents at the cancer tissue site and the distribution of site-specific gene modifications in order to enable the introduction of foreign DNA material into the host genome to destroy tumours [8]. However, some challenges such as the cost and systemic toxicity of these modalities may restrain their application [8]. Thus, it is necessary to focus on novel alternative therapeutic interventions that are non-invasive, with high selectivity for tumour cells and minimal toxicity [9]. 

To address some of the drawbacks that are associated with conventional CRC therapies, photodynamic therapy (PDT) has emerged as a novel alternative intervention in CRC anticancer therapy [5,9]. It is based on the selective uptake of a photosensitizer (PS) molecule followed by PS activation using light irradiation at an appropriate wavelength and as a result, reactive oxygen species (ROS) are produced which in turn lead to cell death [10]. Since PSs are activated only in the light-irradiated area, this allows for more specific targeting of the tumour area compared to systemic chemotherapeutic approach [11].

Although there has been growing interest in PDT applications in the research field, there are still areas for improvement within this novel therapy [12]. To improve the overall efficacy of PDT, it is vital to deliver the PS to the desired cancerous cells and potentially reduce undesirable side effects in normal tissues [13]. With the introduction of the nanotechnology concept to colorectal cancer treatment, the efficient delivery of PSs is an achievable task in PDT advancement [13]. Recently, nanoparticles (NPs) have become well-appreciated owing to their unique properties to potentially facilitate optimal site-specific PS delivery and accumulation through passive or active targeting approaches [14]. 

Most CRC PDT preclinical studies have been conducted on two-dimensional (2D) culture systems and animal models in order to evaluate photodynamic efficiency [15]. Although 2D cultures can provide an understanding of the PS mechanisms of action and cellular responses, there are several setbacks associated with these models [16]. For example, the cells are grown in monolayers which offer unnatural physiological conditions, and there is a reduction in cell-to-cell and cell-to-extracellular matrix (ECM) interactions; therefore, the models fail to capture the realistic presentation of the microenvironment of the cells [16]. The introduction of three-dimensional (3D) culture models in PDT has gained considerable interest over 2D models as they can accurately mimic the tumour microenvironment [16,17]. This review outlines the progress of PDT and the application of nanocarriers as an innovative therapeutic strategy for the effective PDT treatment of colorectal cancer. Additionally, this review highlights the importance of 3D culture models over 2D models in CRC PDT anticancer therapy.

## 2. PDT in CRC Therapeutics

Although there has been great improvement in the development of conventional therapeutic strategies, some of these modalities, such as systemic chemotherapy, lack selectivity and have adverse side effects that still hamper their successful response outcomes in CRC [18,19]. PDT is a tumour-selective and minimally invasive CRC therapeutic modality that utilizes visible light and a photoactivable photosensitizer (PS) to treat CRC tumours [20]. The PS is topically or systemically administered to the cancer site in order to obliterate diseased tissues [10]. After irradiation with a dose of light at a specific wavelength, the PS generates highly cytotoxic reactive oxygen species (ROS) that are capable of initiating oxidative cell destruction and inducing cell death through the mechanisms of necrosis, apoptosis, and autophagy [21,22].

Individually, each element that is required for PDT is non-toxic, however, when the light-sensitive PS is combined with the appropriate wavelength of light and molecular oxygen, the photochemical mechanism results in the production of toxic ROS species that initiate irreversible cell damage [23]. There are several factors that determine the extent of this damage and the mode of cell death that is induced by PDT, including the PS type, subcellular localisation, wavelength and intensity of the light applied, and the type of tumour [10,12].

As an antitumour modality, PDT is considered to be a more localized form of intervention that is highly selective to the targeted cancer cells compared to other CRC treatments such as chemotherapy and radiotherapy [24]. The localized light irradiation in PDT can cause negligible collateral damage to healthy cells with minimal systemic toxicity, as opposed to surgery, chemotherapy, and radiotherapy, which induce systemic toxicity and damage to surrounding cells [22]. As such, PDT can be used as a repeatable protocol since it is generally well-tolerated [25]. Moreover, PDT treatment in comparison to conventional modalities is a simple procedure that can significantly reduce the CRC long-term morbidity [11].

## 3. Principle of PDT

**Figure 2:**
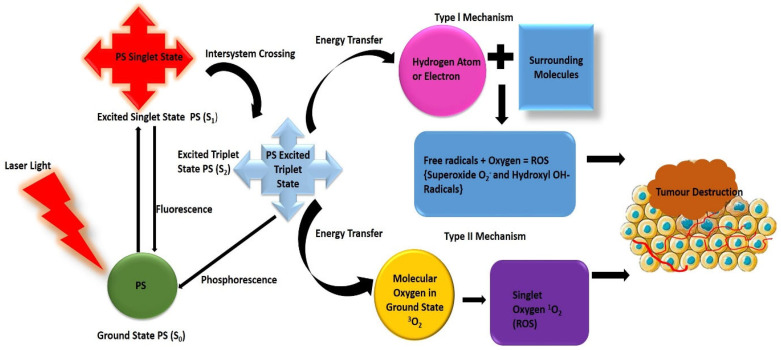
Graphical presentation of photodynamic therapy (PDT) mechanism. Created based on the literature references [10,14].

After exposure to light irradiation at the appropriate wavelength, a photosensitive photosensitizer (PS) absorbs a photon that causes its excitation from its ground state level (S_0_) to a more energetic state called the singlet excited state (S_1_) [26]. The PS then undergoes intersystem crossing (ISC) to the photoactive excited triplet state (T1) [20]. The PS in the excited triplet state (T1) can interact with surrounding oxygen molecules to kill cancer cells through type I or II mechanisms [20]. In the type I mechanism, the PS reacts directly with cell biomolecules and undergoes electron transfer reactions, forming several types of ROS such as superoxide and hydroxyl radicals, which are cytotoxic to the biomolecules [12]. As for the type II mechanism, the excess energy of the PS that is generated in the excited triplet state (T1) is transferred to oxygen molecules, thereby producing cytotoxic singlet oxygen [12]. Consequently, singlet oxygen induces cell death, vascular shutdown, activation of antitumour immune responses, and irreversible damage (Figure 2) [10,12]. Additionally, the phototoxic damage that is exerted by ROS on the neoplastic cell membrane can provoke an inflammatory response to promote antitumour immune responses [27].

## 4. Cell Death Mechanism Associated with PDT 

The reactive oxygen species (ROS) that are formed by the activation of a PS upon light excitation can cause cell damage to intracellular organelles of the cancerous cells and consequently trigger the PDT cancer cell death [23]. Normally, PSs can localize within various cellular organelles, such as the mitochondria, endoplasmic reticulum (ER), Golgi apparatus, lysosomes and plasma membrane [28]. Generally, the mode and extent of the cell death in the targeted cells after PDT depends on the subcellular localisation of the PS, the bioavailability of PS, the concentration of oxygen, the physicochemical properties of the PS, the cell type and the fluency and wavelength of light [23,29]. 

Cellular death via PDT is generally achieved through three different pathways: apoptosis, necrosis, and autophagy [30]. Apoptosis is defined as programmed cell death that is characterized by the energy-dependent biochemical mechanisms of the highly regulated cellular reactions that take place in every cell of the body [30]. There are two main initiating pathways that are involved in the apoptosis process: the activation of death receptors (the extrinsic pathway) or the release of cytochrome c from mitochondria (the intrinsic pathway) [26]. Mitochondria are central processing organelles that play a critical role in the regulation of apoptosis and the energy metabolism of cells; their targeting is highly desirable in order to improve PDT potency [31]. Therefore, the PSs that target the mitochondria or ER can cause photodamage to the mitochondrial outer membrane that is linked to the anti-apoptotic proteins Bcl-2 and Bcl-xL [26,31]. Evidence of apoptotic responses and mitochondrial localisation patterns were reported in the zinc (Zn) metal-based phthalocyanine (ZnPcSmix) PS-mediated PDT treatment of colon adenocarcinoma cells (DLD-1 and Caco-2) [32,33].

Autophagy is thought to be a regulated pathway that is strongly associated with lysosomal degradation of cytoplasmic organelles or cytosolic components [30,34]. This pathway is characterized by double membrane vesicles known as autophagosomes which transport degraded cytoplasmic constituents to the lysosome [11]. Autophagy is critically involved in cellular homeostasis and cell survival, along with the continuous turnover of cellular components through the degradation of cytoplasmic components, and the recycling of the redundant products [30,34]. Although autophagy has been considered as a cell survival mechanism, evidence relating to the autophagy-induced cell death in PDT application continues to accrue [34,35]. Moreover, inhibition of autophagy could serve as a potential avenue for PDT-mediated anticancer therapy [34,35]. Song et al [34] reported that m-tetra (hydroxyphenyl)chlorin (m-THPC)-PDT treatment induced apoptosis and autophagy in CRC cells through the upregulation of ROS, activation of the JNK pathway and inhibition of the mTOR/p70S6K pathway [34]. The inactivation of the ROS/JNK signalling pathway and/or inhibition of PDT-induced autophagy could enhance the efficacy of PDT-mediated treatment in CRC cells [34,35]. The studies went further to demonstrate the significant phototoxicity, inhibited proliferation and increased ROS production that was induced by m-THPC-PDT treatment in HCT116 and SW480 cells [34]. Other studies also suggested that the PS targeting of organelles such as the lysosomes, which have pertinent functions in autophagy, could provoke lethal photodamage of cells and enhance the efficacy of PDT [30,36].

Necrosis is a death mode that involves extensive cell injury and results in irreversible changes to intracellular organelles (the nucleus, mitochondria) [30]. The end-stage of necrosis in PDT is generally characterized by cellular swelling, cytoplasmic granulation, and plasma membrane breakdown [29,30]. In PDT, the mediation of the uncontrolled necrotic death pathway often occurs after an overdose of PS/photoirradiation or direct photodamage of the plasma membrane, resulting in the rapid loss of plasma membrane integrity and cell lysis [30].

In addition to eliciting direct tumour cell death via excessive ROS generation, PDT-induced tumour destruction may also result in the disruption of the tumour microvasculature, thereby depriving the tumour tissue of oxygen and nutrients, resulting in tumour death [10]. PDT is also capable of triggering an inflammatory response, which causes the infiltration of leukocytes to the target area and the release of pro-inflammatory factors and cytokines [37]. Moreover, ROS can further elicit immunogenic cell death, which is accompanied by the release of damage-associated molecular patterns (DAMPs) from the the damaged/dying cells, which then act as danger signals [37,38]. Upon the recognition of DAMPs, the innate immune phagocytes (macrophages, neutrophils and dendritic cells) can neutralize and remove cellular debris [37]. Furthermore, antigen-presenting cells, such as dendritic cells, can be activated (upon stimulation by the DAMPs) and present the tumour-associated antigens (TAAs) and antigenic peptides to the naive T cells, thereby initiating an adaptive immune response, which could provide long-term systemic antitumour immune control [37,39].

## 5. Photosensitizers Used in CRC

PSs are usually non-toxic molecules which absorb visible light at a specific wavelength and, preferably, display a high molecular absorption coefficient [21]. Most importantly, in order to achieve the best PDT efficacy, choosing a suitable PS is one of the most crucial stages [10]. An ideal PS should be chemically pure and highly photochemically and photophysically stable with negligible dark cytotoxicity, and be highly selective for tumours with minimal photosensitivity after excitation [21]. Moreover, an ideal PS should exhibit a high molecular absorption coefficient, with a good absorption in the therapeutic window between 650 and 780 nm (red spectrum of light), in order to penetrate the tissues deeper than 5–10 mm of the targeted tumour area [5,12]. Additionally, it should produce a higher quantum yield in the excited state for ROS generation [10,30]. There are several PSs that are most commonly used for CRC PDT, such as phthalocyanine, porphyrin derivatives, meso-substituted derivatives, chlorin, and hypericin [40]. Generally, PSs for PDT are categorized into three different generations: first-generation, second-generation, and third-generation PSs, some of which have been investigated for CRC therapy and are summarized in Table 1.

### 5.1. First-Generation PSs

PDT first began with the discovery of first-generation PSs, hematoporphyrins (Hp), in the 19th century [25]. A hematoporphyrin derivative, which was formed after the purification of Hp, was the first PS to be clinically approved for PDT [25]. The commercial form of the hematoporphyrin derivative (HpD) is photofrin, which has been widely investigated in PDT treatment of lung, brain, laryngeal, skin, gastric, and esophageal cancers [11]. Sun and colleagues [41] reported the PDT effects and antitumour efficacy of photofrin in patients with advanced CRC [41]. Several other studies have also established that photofrin application in CRC can be an effective PS PDT antitumour therapeutic [25,42].

However, first-generation PSs have been associated with distinct limitations, including: a relatively weak absorption of light and poor light penetration, which is attributed to short wavelength absorption, poor solubility, which can cause photosensitivity, and an undesirable toxicity profile [25]. Moreover, they suffer from poor chemical purity [43]. Consequently, second-generation PSs were developed as a major effort to resolve the shortcomings of first-generation PSs [12].

### 5.2. Second-Generation PSs

In comparison to first-generation PSs, the second-generation PSs possess significantly improved spectral and photochemical characteristics, which are attributed to their structure and composition [44,45]. They have a longer wavelength absorption in the red and NIR region of the spectrum (650–800 nm) and can be used to improve penetration into deep seated tissues [11,25]. In addition, the photosensitivity, stability and tissue selectivity have also been significantly improved compared to first-generation PSs [36,37].

Most second-generation PSs are represented by a group of porphyrinoid compounds that encompass porphyrin or porphyrin-based macrocyclic structures such as benzoporphyrins, purpurins, texaphyrins, phthalocyanines, naphthalocyanines, and protoporphyrin IX [46]. One commonly used second-generation PS, 5-aminolevulinic acid (ALA), which is a precursor of porphyrin, has been extensively studied over the years and has demonstrated considerable potential in PDT and photodiagnosis for CRC treatment [7,40,46].

The phthalocynanine types of PSs such as zinc phthalocyanine (ZnPc), aluminium phthalocyanine tetrasulfonate (ALPcS_4_), and silicon phthalocynanine (HOSiPcOSi(CH3)_2_(CH2)_3_ N(CH3)_2_ (Pc4), and the chlorin-structured PSs such as monoaspartyl chlorin e6 (NPe6) and temoporfin are other commonly used second-generation PSs in CRC treatment that have demonstrated tremendous potential efficacy [40,47,48]. This is mostly due to their improved photophysical characteristics such as a high absorption coefficient in the red to near-infrared spectral region up to ~750 nm, thereby offering a great depth of penetration and the generation of a high quantum yield of singlet oxygen [40,49]. Furthermore, in addition to their absorption Q bands in the red region, sharp Soret bands have been observed for some of the second-generation PSs that were derived from porphyrins such as zinc phthalocyanine and chlorin e6 (Ce6) [40,50], which could offer diagnostic opportunities.

Li et al. [51] reported the improved phototherapeutic effect of PDT when chlorin e6 (Ce6) was used [48]. This second-generation Ce6 PS demonstrated improved ROS generation, enhanced apoptosis, inhibition of cell proliferation and an overall high PDT sensitising efficacy in SW480 colon cancer cells after laser-light irradiation using a 650 nm laser, at an irradiation dose of 6 J/cm^2^ [51]. Similarly, the use of phthalocyanines has been reported to be effective in CRC treatment, particularly those that were modified by the co-ordination of transition metal ions such as aluminium and zinc [40]. They have been extensively investigated in CRC PDT because they exhibited high singlet oxygen quantum yields and displayed efficient ROS generation and phototoxicity [40]. Related to CRC PDT treatment, Table 1 summarizes findings from CRC PDT-related studies that utilized first- and second-generation PSs.

Although second-generation PSs have demonstrated great potential in PDT, they suffer from several drawbacks such as poor solubility in aqueous solution and poor tumour selectivity, which not only affect the uptake of the PSs, but also their subcellular distribution [45]. For the further improvement of PDT efficacy, third-generation PSs were introduced, which are composed of second-generation PSs that are conjugated to nanoparticle carriers, and antibodies [24]. These PSs have a higher selectivity and specificity for the cancerous tissues [14,45].

### 5.3. Third-Generation PSs

The third-generation PSs have been proposed by researchers to improve the bioavailability of PSs and alleviate undesirable systemic side effects, resulting in better therapeutic effects [10]. The application of nanotechnology as a fundamental tool to PDT in CRC is the key research direction to resolve some of the drawbacks that are associated with second-generation PSs and to improve selective cellular localisation in affected tumours [14,24]. Generally, third-generation PSs are comprised of second-generation PSs that are either anchored to or encapsulated by nanocarriers such as nanoparticles, liposomes or micelle carriers in order to increase the uptake and accumulation of NP-PSs by cancerous tissues [18,52]. The NPs can also be decorated with active targeting agents such as antibodies, biomarkers, or ligands in order to efficiently bind to receptors that are overexpressed on the surfaces of cancer cells [5,18]. This new generation of PSs has displayed a longer absorption spectra and exhibited improved characteristics such as an increased biocompatibility, enhanced cancer targeting capabilities, and increased levels of ROS production relative to the first- and second-generation PSs [53].

**Table 1:** First- and second-generation photosensitizers evaluated in pre-clinical studies for CRC PDT.

PS	Generation	Cell Type	Remarks	Ref.
Sinoporphyrin sodium and photofrin	2nd, 1st	HCT-8 and HCT-116	The effects of sinoporphyrin sodium-PDT and photofrin-PDT resulted in significant antitumour efficacy	[54]
Tetraaryl brominated porphyrin (TBr4) and with the diaryl (BBr2) derivative.	2nd	Colorectal adenocarcinoma cells, HT29	Significant reduction in cell growth and necrotic cell death within in vitro and in vivo studies	[55]
Gallium (III) phthalocyanine chloride (GaPcCl)	2nd	Caco-2	GaPcCl with PDT led to 60% to 80% cell viability cytotoxic and apoptotic cell death.	[56]
Tetra 4-(3-(piperidinium-1-ylmethyl) phenoxy substituted zinc (II) phthalocyanine (Zn6a)	2nd	colorectal carcinoma (HCT-116)	High phototoxicity on HCT-116 cells	[57]
Selenium tetrasubstituted zinc (II) phthalocyanines	2nd	Murine colon carcinoma CT26	Significant increment in ROS level and efficient antitumour effect.	[58]
Hypericin (HY)	2nd	SW480 and SW620	HY mediated PDT demonstrated cytotoxic effect and inhibition of tumour cell proliferation in a dose-dependent manner.	[59]
Chlorin e6 (Ce6)	2nd	SW620	Ce6 mediated PDT significantly reduced the healing and migration rate of colon cells.	[60]
5-aminolevulinic acid	2nd	SW480 and SW620	PDT with 5-ALA improved anticancer effects and inhibited of the secretion of cytokines (IL-10)	[61]
Ce6	2nd	SW480	Decreased cell survival rate in a dose-dependent manner and significant inhibitory effect on F-actin microfilament and cytoskeleton.	[62]
5-aminolevulinic acid	2nd	Caco-2	Cell viability inhibition~62.4%, and improved antitumour efficacy	[63]
5,10,15,20-Tetra(quinolin-2-yl) porphyrin (2TQP)	2nd	HT29 colorectal adenocarcinoma	2-TQP displayed effective phototoxic effects with no dark toxicity on cells	[64]
Hypericin (HYP)	2nd	HCT116 and SW620	Cell proliferation inhibited, and efficient ROS generated by HYP-PDT treatment. Apoptosis was induced	[65]
Sinoporphyrin sodium (DVDMS)	2nd	CX-1	DVDMS-PDT triggered apoptosis. Inhibitory effect in a dose and time dependent manner	[66]
				

## 6. Current Limitations of CRC PDT

Although PDT has been reported to be an effective alternative therapy for CRC treatment, it still has certain drawbacks such as poor PS water solubility, the difficulty of treating deep-seated tumours due to the low tissue penetration of the illuminating light, and the difficulty of PS localisation at depths at which cancerous tissues can be targeted [5,18]. The common PSs, particularly the first-generation PSs, are often associated with extensive retention within cancer tissue, which can to lead to skin photosensitivity [11]. In traditional PDT, high doses of unconventional PSs within the cancerous tissues are often required, which can cause systemic toxicity and serious damage to healthy tissues [67].

Moreover, cancer tissues are generally in a low oxygen state, and therefore provide an insufficient oxygen yield for the overall efficacy of PDT, resulting in PDT-induced hypoxia which compromises the generation of ROS and the complete destruction of the tumour [5]. Additionally, most of the traditional PSs, such as porphyrins and other tetrapyrrole derivatives, are poorly soluble in physiological solutions; thus, they have a strong tendency to aggregate, which could hinder their bioavailability and biodistribution within tumour cells [68].

Conventional PDT, like other traditional therapies, has also been associated with recurrence and resistance [69]. The possible mechanism of resistance to PDT may be attributed to several properties, such as inherent tumour heterogeneity and drug efflux [69,70]. Nonetheless, studies have reported that a cancer cell population that is resistant to chemotherapeutic treatment, to some extent, can be slightly more susceptible to PDT [70]. The recurrence and progression of colorectal cancer (CRC) has been linked to the presence of cancer stem-like cells (CSCs) that exhibit a high resistance to PDT [35]. Therefore, therapeutic strategies might be required in order to eliminate the advanced types of CRC, including both the primary tumours and the secondary systemic disease [5].

In this regard, extensive investigations into the application of nanoparticle (NP)-based PS drug carriers have been pursued with the hope of providing an alternative that minimizes some of the shortfalls of conventional PDT by improving PS uptake within the CRC cells [14,18]. For this purpose, several nanocarrier platforms such as liposomes, dendrimers, and polymeric and inorganic nanoparticles have been developed and modified with PSs in order to minimize the systemic side effects and increase the therapeutic effects of CRC PDT [18,52].

## 7. Nanotechnology as a Favourable Strategy in PDT for CRC Therapy

The application of NPs in CRC PDT treatment presents a great potential to alleviate several of the limitations of traditional PSs and increase their bioavailability [24,43]. NPs are distinct nanocarriers that could enhance the efficient delivery of PS molecules to targeted sites and minimize the therapeutic side effects, thereby increasing PDT effectiveness [14]. NPs are typically between 1 and 100 nm in size [24] and they generally exhibit a relatively large surface-area-to-volume ratio, which can increase their surface interaction with PSs and promote the loading capacity of the PS, thereby improving the concentration delivery and enhancing either passive or active uptake in the cancerous cells [71,72]. The controlled small size range of NPs offers accurate mimicking of biological molecules, protection of the PS cargo from the hostile immune system barriers, and prolongation of the PS systemic circulation lifetime and PS delivery, while avoiding unwanted side effects [72]. Furthermore, NPs with smaller sizes can facilitate the transportation of the PS to the targeted cells by exploiting the enhanced permeability and retention (EPR) effect [14]. 

NP carrier platforms can also be fabricated to exhibit favourable distinct physicochemical and biological properties by altering their composition, shape, size, and surface properties [72]. In addition, they are favourable platforms for PS delivery owing to the easy encapsulation or embedment of PSs, as well as various other active moieties [24]. Functionalisation of NPs with active moieties offers better stability and solubility, reduced toxicity, improved localized PS delivery, enhanced selectivity and site-specific PS delivery to targeted cells, thereby ultimately improving the PDT therapeutic efficiency [22,72].

### 7.1. NPs-Mediated PS Delivery in CRC PDT

The application of NPs in PS delivery has presented multiple benefits in resolving the drawbacks that are associated with traditional PSs. The types of nanocarrier delivery platforms that have been employed as carriers of PSs in CRC PDT can be categorized into organic nanocarriers such as liposomes, lipid NPs, and polymeric NPs, and inorganic NPs that include silver, gold, quantum dots, and silica nanoparticles [24,73]. Among organic NPs, liposomes are comprised of an aqueous core enclosed by phospholipid bilayers which can encapsulate the PS for efficient delivery [24,73]. They also display several advantages such as biocompatibility and biodegradability characteristics, and a high loading capacity to carry the PSs. They also protect PSs from early degradation and environmental factors [73]. In one study, Bakhshizadeh et al. [74] investigated the PDT/sonodynamic therapy (SDT) effect of liposomes that were incorporated with zinc phthalocyanine against a CT26 cell line that was derived from a colon tumour carcinoma of a BALB/c mouse [74]. The final liposomal zinc phthalocyanine formulation presented an average size of 40 nm with a great encapsulation efficiency of more than 85% [74]. Moreover, liposome-encapsulated zinc phthalocyanine significantly inhibited the growth of the CT26 tumour in comparison to the untreated control groups within the sonophotodynamic therapy (80% of tumours were recovered) [74]. According to the results, the zinc phthalocyanine incorporated in the liposome that is associated with SPDT treatment may possibly be an effective treatment for CRC [74]. Likewise, Wu et al. [75] represented a novel drug-delivery system of m-THPC PS, FosPeg^®^ which was a new liposomal formulation of m-THPC [75]. Improved PS absorption and PDT antitumour effects were observed in the HT-29 human colorectal adenocarcinoma cell line [75]. The liposomal formulation most likely improved the delivery of the PS [73,76].

Inorganic NPs have also presented several advantages in CRC PDT [5,18]. The noble metallic NPs such as gold NPs (AuNPs) have high surface-to-volume ratios and are easily tuneable, which offers the possibility of functionalize them with antibodies [72]. They also have a low toxicity with negligible side effects [72]. Additionally, gold NPs display inherent physicochemical properties such as the surface plasmon resonance (SPR) that could introduce heat or toxic radicals into the tumour tissues after the irradiation during PDT [77]. PEGylated gold NPs also present good biocompatibility with the biological system, so they can passively accumulate within tumours through the EPR effect [14,77,78]. A study exploring AuNPs for use in targeted PDT in CRC was proposed by Obaid et al. [79]. The authors stabilized AuNPs with a mixed monolayer of the hydrophobic ZnPc photosensitizer (C11Pc) and the hydrophilic polyethylene glycol (PEG), and further covalently bound them to jacalin or monoclonal anti-HER-2 antibodies [79]. The zinc phthalocyanine-gold NPs with antibody moieties demonstrated an increased phototoxicity in HT-29 colorectal adenocarcinoma cells [79]. Thus, the nanoconjugate was able to selectively kill targeted HT-29 colorectal adenocarcinoma cells via PDT [79]. 

**Figure 3:**
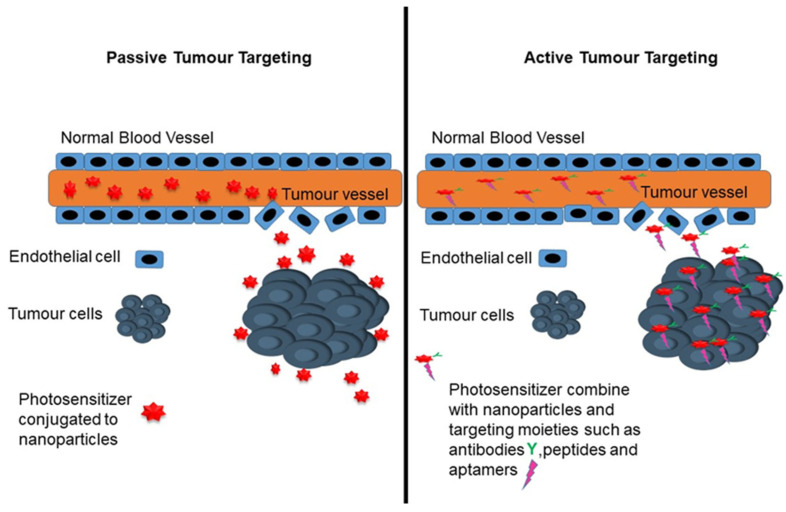
Schematic presentation of passive and active tumour targeting.

There are two strategies that are most commonly used to mediate the targeted delivery of PSs to tumours using NPs: passive and active targeting strategies, which are illustrated in Figure 3 [8,69].

#### 7.1.1. Passive Targeting Strategy

Passive targeting generally exploits the enhanced permeability and retention (EPR) effect in order to facilitate the effective accumulation and delivery of PS nanocarriers in the tumour cells [20]. Passive targeting of NP-PS occurs due to the different pathophysiological characteristics of the solid tumour, which can favour the accumulation of nanocarriers [80]. Generally, a leaky tumour vasculature and the poor lymphatic drainage due to the abrupt neovascularisation that is associated with rapid cancer growth can determine the extent of the EPR effect [80]. The EPR effect can compromise the microenvironment within the CRC tumours, allowing the permeation and accumulation of NP-PS at the cancerous site [80]. Therefore, intravenously administered NP-PS would tend to selectively accumulate more in the CRC cancerous tissues, relative to healthy tissues, and relatively improve the activity of the NP-PS at colonic tissues [80]. 

Recently, numerous in vitro and in vivo studies have demonstrated the merits of using passively targeted NP-PS approaches against colorectal cancer [18]. In one study, Yurt and colleagues [81] introduced titanium dioxide NPs (TiO_2_ NPs) that were loaded with subphthalocyanine (SubPc) PSs [81]. During the in vitro study, the authors found that the SubPc-TiO_2_ nanoparticles had superior efficacy and exerted higher phototoxicity on PDT-treated HT29 colon cells compared to cells that were treated with free SubPc [81], which improved the cellular internalisation of the nanoconjugates by passively targeting the NPs [81]. The SubPc-TiO_2_ also displayed theranostic/fluorescent potential in colon tumours [74]. In another study, de Freitas et al. [82] investigated the cellular uptake of curcumin (CUR) that were combined with silver nanoparticles (AgNPs) and hydrogels comprised of chitosan (CHT) and chondroitin sulphate (CS), which are natural biopolymers from the vitro study of Caco-2 cells [82]. The CHT/CS/CUR-AgNPs yielded significant Caco-2 inhibition after PDT photoactivation [82]. In addition, the fluorescence intensity of curcumin on the Caco-2 cells that were treated with the nanoformulation increased owing to the EPR effect, which most likely improved the preferentially cellular absorption of CUR [82]. Similarly, Ballestri et al. [83] improved the passive tumour-targeting of non-symmetric di-methyl-amino-ethylacrylate diaryl-porphyrin (PorVa) PSs that were conjugated with core-shell poly methyl methacrylate NPs (PMMA) [83]. They showed that PSs conjugated with PMMA NPs improved antitumour effects and efficiently eliminated cancer, upon irradiation of an in vitro-cultured HCT116 human colon carcinoma cell line [83]. Studies have reported that the size and shape of the NPs that are utilized in PDT can generally affect their interactions with biological barriers and tumour microenvironments, and subsequently compromise their accumulation via the EPR effect [84] In this sense, smaller sized NP-PS delivery vessels are possibly desirable in order to further improve the penetration of PSs into tumours [48]. Moreover, the introduction of modifications to the surface of the nanoparticles, such as polyethylene glycol (PEG) could shield them from opsonisation and aggregation and prolong their circulation time [69,70]. Polyethylene glycol (PEG) is one of the commonly utilized ligands in the designing of multifunctional NPs for PS drug delivery [69,70]. For instance, Ryu et al. [85] reported the use of nanophotosensitisers that were composed of methoxy poly(ethylene glycol) (MePEG) and Ce6 with a small diameter of less than 100 nm in the Ce6-mediated PDT treatment of colon cancer cells [85]. The Ce6 nanophotosensitisers demonstrated an improved cellular uptake, phototoxicity, and reactive oxygen species (ROS) generation in the in vitro cell culture experiment [79]. Additionally, the Ce6 nanophotosensitiser nanoconjugates could selectively accumulate in CRC tumours and ethoxy poly(ethylene glycol) (MePEG) could enhance the tumour tissue penetration [85].

Within the subject of in vivo experiments, several studies have also evaluated the advantages of passive targeting in CRC PDT [15]. For example, Bretin et al. [86] studied the photodynamic activity of a fabricated PS 5-(4-hydroxyphenyl)-10,15, 20-triphenylporphyrin (TPPOH) xylan-coated silica NP (SNP) within CRC xenograft mice models [86]. The SNP/TPPOH-mediated CRC PDT treatment presented a dramatically improved uptake and sufficient ROS production which led to increased cytotoxicity and improved PDT anticancer efficacy on human CRC cell lines [86]. They suggested that the improved uptake was probably attributed to passive targeting via the EPR effect [86]. Table 2 highlights in vivo studies demonstrating the potential of passive targeting of NPs to achieve improved PDT efficacy within CRC treatment.

#### 7.1.2. Active Targeting Strategy

The active targeting strategy involves the use of high-affinity ligands or targeting moieties on the surface of the nanocarriers that bind to specific overexpressed receptors on the target tumour cells, in order to enhance the overall PS cellular uptake and localisation [14]. Moreover, the use of targeting moieties could possibly enable the delivery of the PS at the targeted site, which could significantly increase their cytotoxic effect and reduce unwanted side effects [14,18].

The surface of NPs can be functionalised with targeting moieties such as aptamers, ligands, monoclonal antibodies, and antibody fragments that could identify specific cancer cell receptors in order to promote specific targeting [14].

Some of the widely investigated receptors and possible targets for CRC PDT include the epidermal growth factor receptor (EGFR), transferrin receptors, fibroblast growth factor receptors (FGFR), and the epithelial cell-adhesion molecule (EpCAM), among others [13]. For example, the transferrin receptor (CD71) is generally expressed to regulate iron homeostasis within normal human cells, however, malignant tumours are often found to express abnormal levels of expression of the transferrin receptor (CD71), which may be targeted by antibodies in targeted PDT [92]. In one study, Sardoiwala et al. [92] synthesised hypericin-loaded transferrin nanoformulations (HTfNPs) for the treatment of colorectal cancer [92]. The nanoformulation demonstrated stability and when it was used in PDT, it showed efficient generation of cytotoxic reactive oxygen species (ROS) [92]. Additionally, the anticancer effect of HTfNP-assisted PDT via the induction of PP2A-mediated BMI1 was demonstrated [92]. Moreover, the transferrin nanoparticles demonstrated the improved bioavailability of hypericin and better targeting at the tumour site [92]. In another study, Wang et al. [93] proposed a transferrin-IR780 NP (Tf-IR780 NPs) system for use on transferrin-overexpressed CT26 tumours in both in vitro and in vivo studies in order to actively target and suppress tumours [93]. This nanosystem showed improved targeting and greater antitumour effects, suggesting that the PS nanoconjugates had specifically targeted the transferrin receptors that were overexpressed on CRC cells [93]. 

Antibody-mediated NP-PS delivery is being investigated for PDT active targeting and may feasibly reduce the unwanted side effects in healthy cells as well as enhance targeting efficiency, owing to the high specificity and affinity of the antibody–antigen interactions [84]. One of the desirable properties of the targeting moieties that are conjugated onto the NP is their high affinity as well as their capability to specifically recognize and actively bind to the appropriate antigens or receptors that are exclusively or uniquely overexpressed on only the targeted CRC cells [5]. Subsequently, NP-PS internalisation through receptor-mediated endocytosis is improved and PS delivery is enhanced [94]. Table 3 summarizes the studies that investigated active PS delivery in CRC.

## 8. PDT Combined with Other Therapies in CRC Treatment

It is well documented that in colorectal tumours there is evidence of complex heterogeneity within specific mutations, which may present challenges for the majority of current treatment modalities [101]. In general, several conventional monotherapies that are used in CRC anticancer treatment have presented relatively unsatisfying outcomes in terms of the complete eradication of CRC cells, and they also result in the development of unwanted side effects [5]. Establishing combinations of synergistic therapies has considerable appeal owing to their many merits over single treatment, including their improved efficacy by synergistic effects and their reduced side effects [5]. The following section gives an overview of targeted PDT in combination with two other therapies, among others, in an effort to enhance efficacy, while overcoming undesirable side effects.

PDT is capable of triggering immunogenic cell death, which is a PDT-induced cell death that stimulates immune responses and induces antitumour immunity, so it may be used in combination with immunotherapies that harness and boost the host’s immune system, such as antibodies that block the suppressive immune checkpoint mechanism/immune checkpoint inhibitors [102]. The strategy of using NP-PS formulation in the PDT-PD-L1 checkpoint blockade combinative approach has shown promising therapeutic effects in clinical studies. He et al. [103] conjugated nanoscale co-ordination polymer (NCP) NPs that carried oxaliplatin and pyrolipid PS (NCP@pyrolipid) [98]. The integration of oxaliplatin chemotherapy, PDT, and checkpoint blockade therapy enhanced antitumour immunity and exhibited effective therapeutic effects for the treatment of metastatic colorectal cancer, as well as potentiated the PD-L1 checkpoint blockade [103]. In another study, Xu et al. [104] employed upconversion nanoparticles (UCNPs) that were incorporated with chlorin e6 (Ce6), and imiquimod (R837), which is a Toll-like-receptor-7 agonist [104]. After near-infrared (NIR) irradiation caused a significant response in the phototoxicity rate of the effective primary tumours, death was observed in the CT26 cells [104]. The results suggested that the UCNP-Ce6-R837-based PDT under NIR irradiation is a promising anticancer strategy that can lead to the significant inhibition of distant tumours and the inhibition of tumour relapse [104]. 

The combination of photothermal therapy (PTT) and PDT is also favourable in treating CRC, owing to the cytotoxic ROS and hyperthermia that are generated by PSs under light exposure [105]. Seo et al. [105] studied the PDT/PTT effects of methylene blue that was incorporated into gold nanorod@SiO_2_ (MB-GNR@SiO_2_) core@shell NPs on a CT-26 mouse colon and CT-26 cancer cells [105]. Upon irradiation with a 780 nm wavelength of laser light with a power density of 1 W/cm^2^, the cancer-killing efficacy was significantly enhanced [105]. In a recent study, Wang et al. [106] designed a hyaluronic acid (HA)-polydopamine nanoparticles (PDA-NPS)-chlorin e6 (Ce6) (HA-PDA-Ce6) formulation based on the PDT/PTT cancer targeting therapy [106]. The synergetic effects of the HA–PDA–Ce6 demonstrated an enhanced accumulation within tumours, increased tumour growth inhibition and improved phototoxic effect in HCT-116 tumour-bearing mice [106]. 

From the presented studies it is evident that NP-based PS delivery in PDT and combinative therapies has demonstrated great potential for the treatment of colorectal cancer. Nonetheless, further studies are still required in order to investigate NP-PS effectiveness within CRC, after PDT is combined with other treatment approaches in clinical settings.

## 9. Application of 3D Tumour Models in PDT CRC Treatment

The majority of in vitro PDT CRC studies are based on a two dimensional (2D) cell culture where cells are cultivated as monolayers on flat surfaces, as well as in vivo models [107]. Microscopic and molecular studies are made easier by 2D monolayer cultures, and these models also offer several benefits, including easy preparation, maintenance, and monitoring [16]. However, the current 2D models fail to adequately integrate the interactions between the cells and the surrounding extracellular matrix (ECM). Moreover, the environment of 2D models cannot mimic the characteristics of a tumour in human physiological environments [16]. Therefore, in order to better evaluate the CRC cellular response to PDT as well as optimize the physiological resemblance between in vitro models and the human environment, and the heterogeneity similarity of tumours, 3D tumour models have attracted considerable interest [108]. These models can improve the accuracy compared to traditional 2D cell cultures, while still possessing high throughput relative to in vivo models [108]. 

The use of several 3D tumour culture models, such as scaffold-based platforms, microfluidic platforms, and multicellular tumour spheroids (MCTS) has led to improvements in CRC photodynamic studies on the uptake of PSs [16]. Khot et al. [109] noted the cellular response variations between 2D and 3D models of CRC, after PDT treatment [109]. For this purpose, spheroids were formed using forced-floating and agitation-based techniques, and HCT116 and HT29 CRC cells were treated with hypericin for 16 h under 1 J/cm^2^ of fluence [109]. Interestingly, the 3D spheroid models demonstrated more resistance towards hypericin-mediated PDT compared to 2D models, which may be attributable to the upregulation of ABCG2 [109]. These results exemplify the benefits of the 3D structures, which mimic the physiological conditions and can be valuable in obtaining insightful strategies and an understanding of the mechanisms that are important for in vivo pre-clinical studies [109]. Similarly, studies reported the effects of methyl 5-aminolevulinic acid-PDT using a light emitting diode in 2D and 3D models of Caco-2 and SW480 CRC cells [110]. Caco-2 spheroids were grown for three days using the liquid overlay technique and SW480 spheroids were produced from the hanging drop method [110]. The results showed that after methyl-5-aminolevulinic acid (Me-ALA)-PDT treatment, the Caco-2 spheroid mass was inhibited [110]. Additionally, it was observed that, even though the spheroid mass dimensions were reduced, the spheroids were significantly resistant to PDT in comparison to those cells that were grown on 2D monolayers. [110]. It was suggested that these effects was initiated by hypoxia as the main photodynamic block within the spheroids [110].

Among the 3D tumour culture models, MCTS spheroid cultures in CRC PDT have particularly attracted extensive research consideration because they retain the 3D architecture that contains the ECM distribution and have characteristics of human tumour environments [16]. Additionally, many features of 3D models mimic the microenvironment found within tumours in vivo, such as the low oxygen gradient, deposition of extracellular matrix, pH, hypoxia and necrotic cores [17]. Generally, once MCTs spheroids are sufficiently large enough, they display the characteristic features of a vascular tumour: a proliferative zone, an inner quiescent zone and a necrotic zone [17,111]. The outer proliferative zone is comprised of cells that receive sufficient oxygen and other nutrients that are required for proliferation [17,111]. Whereas inside the spheroid, an inner quiescent zone and a necrotic core are formed [111]. Within the inner quiescent zone, the cells remain viable; however, they do not proliferate [111]. In the necrotic zone, the innermost cells die due to the deprivation of the oxygen and nutrient supply, as well as the toxic waste from accumulated products [111].

MCTS spheroid culture models are simple enough to grow and can generate large quantities of 3D spheres at minimal costs [16]. Thus, they are highly sought for PDT studies that investigate PS uptake, mechanisms of PDT therapeutic interaction and efficacy, and that evaluate the penetration capacity of PDT anticancer therapeutic approaches [17]. For instance, Pereira et al. [112] reported the phototoxic effects of porphyrin that was conjugated with four glucose molecules (PorGlu_4_) in both monolayer and spheroid cultures of HCT-116 colon cancer cells [112]. Within the spheroid models, HCT-116 colon cancer cells expressed decreased GLUT1 protein levels, which increased the endogenous ROS [112]. Subsequently, the ROS-induced phototoxicity in spheroids after PorGlu_4_-mediated PDT treatment [112].

In nanoparticle-mediated PDT studies, 3D models have also shown improved photodynamic effects [113,114]. For example, Gibot and colleagues [114] reported the photodynamic activity of a PS, pheophorbide (Pheo) that was encapsulated with various polymeric micelles, poly(ethyleneoxide-b-ε-caprolactone), poly(ethyleneoxide-b-d,l-lactide), and poly(ethyleneoxide-b-styrene), in 2D and 3D tumour spheroids [114]. The authors showed that the encapsulation of Pheo with the suggested polymers increased photocytotoxicity [114]. The authors also presented that using PS drug-delivery systems based on the copolymer-based formulations showed an effective delivery of the Pheo in HCT-116 spheroids as well as in 2D cells (delivery efficiency slightly varied amongst the nanovectors) [114]. So, since the PS in the encapsulated state was unlikely to agglomerate, it could probably dissociate in the cancerous cells [114]. Therefore, these nanoformulations are potential candidates for PS delivery and could possibly promote accumulation in the spheroids and effective inhibition of colorectal tumour growth [114]. Another study by Till et al. [113] investigated the efficacy of crosslinked nanovectors as well as the photocytotoxicity effects of the Pheophorbide A (Pheo)-loaded micelles poly(ethyleneoxide-b-3-caprolactone) [113]. 3D spheroids were formed on ultra-low attachment well plates from human colorectal carcinoma (HCT-116) cells [113]. The results demonstrated that the crosslinked nanovectors significantly reduced tumour size by up to 80% at day two and approximately 90% at day four [113]. Lee et al. [115] have also reported a significant reduction in tumour growth in the spheroids of the mouse musculus colon carcinoma (CT26), when liposome nanoparticles that were loaded with a zinc phthalocyanine (ZnPc) platform were utilized [115]. However, even though 3D models have widely attracted attention to anticancer PDT investigation and have been shown to offer a more realistic prediction of the efficiency of PDT treatment in vitro and in vivo, their application in PDT CRC still requires further research.

## 10. Clinical Application of PDT in CRC Treatment

Clinical PDT treatment involves the application of visible light that is combined with a PS and oxygen to destroy CRC cells in patients [7]. PDT treatment has shown to be a successful approach in clinical studies, and several PSs such as ALA, verteporfin, porfimer sodium (Photofrin), temoporfin have now been approved for clinical applications in the treatment of various cancers, including skin cancer, actinic keratosis, myopic choroidal neovascularisation, bladder cancer, and advanced head and neck squamous cell carcinomas [10]. Currently, the majority of PSs that are used in the clinical application of PDT in CRC are Photofrin, ALA, and HpD [15]. Typically, the clinical application of PDT in CRC treatment is performed with endoscope optical fibres in order to deliver the PS and the visible light that is required for its excitation [18]. In this way, PDT shows selective damage only to cancerous colon tissues, with negligible undesirable side effects and minimal systemic cytotoxicity to nearby healthy cells [18]. For instance, Welbourn et al. [116] conducted a clinical pilot study of PDT for invasive anal squamous cell carcinoma in 15 patients, where 12 of these had anal intra-epithelial neoplasia (AIN), 2 patients had intra-epithelial adenocarcinoma and 1 patient had dysplasia with high-risk human papillomavirus [116]. The efficacy and safety of topically applied ALA and systemically administered Photofrin, at a dose of 1.2 mg/kg of body weight, with PDT was investigated in this study [116]. Topical ALA–PDT was applied to the localized lesions with two cycles of a fluence of 37.5 J/cm^2^ laser irradiation, and for systemic PDT, a 630 nm laser with a fluence of 100 J/cm^2^ was delivered [116]. The study reported the complete PDT response of 6 patients that had AIN II or III, and 10 patients that had aceto-white staining [116]. In another study, Sun et al. [41] reported a clinical trial using photofrin photodynamic adjuvant treatment in 23 patients that had advanced colorectal cancer [41]. Photofrin was administered intravenously at a dose of 2 mg/kg in 100 mL of 5% glucose, after 48 hours, and laser irradiation at a 630 nm intensity was delivered with an endoscopic optical fibre [41]. The necrotic tissue biopsies were removed endoscopically from the treatment site several days post-treatment, and repeated irradiations were performed at the cancerous site [41]. After one month post treatment, the patients underwent an enteroscopy examination to assess the therapeutic effect of the therapy [41]. The observation group exhibited significantly higher effectiveness and improved survival rate of the therapy, compared to the control group of 30 patients [41]. Skin photosensitivity was the only minor complication that was observed following laser irradiation [41]. 

The liver is one of the organs to which colorectal cancer most frequently metastasizes [117]. About 15–25% of patients with colorectal cancer generally develop liver metastases at diagnostic stages [117]. Surgical removal of tumours is the first line of treatment; however, it has side effects, as previously mentioned [117]. PDT can be applied as an alternative treatment option for liver metastases, and has displayed satisfactory results [117]. Vogl et al. [118] reported results of PDT in 5 patients (4 women and 1 man), with six liver metastases of colorectal carcinoma [118]. PS SQN 400 (mTHPC) was administered at a dose of 6 mg/kg body weight in three patients and 3 mg/kg body weight in the other two patients [118]. At a time of 120 h later, the PS was activated by a diode laser at a 740 nm wavelength and a fluency of 60 J/cm [118]. It was observed that the tumour was successfully removed in about 60% of the patients [118]. More importantly, several other phase I and phase II clinical studies of PDT in CRC have reported on the safety and effectiveness of the PDT application [42].

With reference to PDT in clinical application for CRC treatment, the presented studies have adequately demonstrated the effectiveness of the modality. However, according to our knowledge, there is currently still a limited amount of published data on phase III-randomized clinical trials and clinical trials of phase IV PDT CRC therapy that have been conducted thus far [42]. Researchers should, therefore, take advantage of nanotechnology applications as an attractive strategy for the enhancement and betterment of PDT, and consider further research in order to propel targeted PDT into more clinical applications.

## 11. Conclusions and Future Perspectives

The effectiveness of PDT in eliminating CRC has been investigated in several in vitro and in vivo clinical trials (although limited), and the outcomes of the studies demonstrated the remarkable potency of PDT with relatively minimal adverse events [62,63,64,65,66]. Despite the promising outcomes, the full potential of PDT is compromised by traditional PSs that impose limitations in terms of poor tumour targeting, insufficient quantum yield, low cellular uptake, and insufficient penetration depth [72]. In this regard, NPs have been highlighted as favourable platforms to enhance the delivery of the PSs into the targeted CRC tumours [5,18]. NPs are potential candidates for PDT since they can possibly assist in navigating through some of the obstacles: they can enhance the bioavailability and solubility of the PSs, the tumour selectivity and specificity with negligible side effects, and the overall enhanced PDT efficacy [72]. Moreover, NPs serve as platforms that promote the passive PS uptake to cancerous cells through the EPR effect, thus further improving the PS cellular uptake [72]. In addition, in order to further enhance tumour selectivity, the PS-loaded nanocarrier systems can be targeted to CRC tumours by modifying the nanosystem with specific ligands that are recognized by the overexpressed receptors on CRC cells [18,72]. Several targeted NP-PS formulations have been developed and demonstrated photodynamic efficacy within in vitro and in vivo in CRC PDT treatment (Table 3).

Although the role of NPs is significantly beneficial in PS delivery and PDT for CRC treatment, it is still the subject for further investigations in terms of developing new designs and novel targeting moieties that can possibly increase specific targeting in PDT. Moreover, even with several studies in in vitro and in vivo NP PDT applications, only a limited number of NPs have been utilized in clinical trials in PDT-CRC treatment. Therefore, there is a further need to improve the delivery of NP-PS, particularly those that are functionalised with site specific ligands in CRC-PDT and their applications for clinical PDT treatment, in order to meet the full potential of the NPs. It should be kept in mind that several passive and active targeting NP-PS strategies are based on in vitro studies and still rely on 2D cell cultures, which may offer a limited insight into in vivo experiments and human trials. 2D cell cultures are generally characterized by an unrealistic environment in which CRC cells are grown in unrealistic conditions that cannot mimic the characteristics of human CRC tumours. Undoubtedly, 3D cell culture models that could resemble the tumour microenvironment are considered as a starting point to understand the factors necessary to mediate effective cell targeting and predict tumour response, especially when used in NP-mediated PDT. Thus, these models are attractive and require extensive investigations.

## Figures and Tables

**Table 2:** Summary of in vivo studies PDT of passive NP-based PS delivery systems in CRC PDT treatment.

In Vivo CRC PDT Studies Reported on Passive Nanoparticle-Based Photosensitizers				
Nanosystem	PS	Key Findings	Cell Type	Ref.
chitosan nanoparticles (CS NPs)	Encaspulated 5-Aminolevulinic acid 5-ALA and photothermal (IR780)	Superior photodynamic cytotoxicity effects, higher tumour accumulation	mouse colon tumours CT-26 cells	[87]
lipid nanoparticles	HPPH	Effective accumulated in colon tumours and enhanced anticancer activity	Murine CT-26 colon carcinoma and HT29 tumour bearing mice	[88]
PheoA-ss-GC nanoparticles (PheoA-ss-CNPs),	pheophorbidea (PheoA)	Increased selective accumulation and significant reduction in tumour growth	HT-29 tumour-bearing mice	[89]
Functionalized polyacrylamide (AFPAA)	2-[1-hexyloxyethyl]-2-devinyl pyropheophorbide-a (HPPH)	Improved localisation and the tumour response to the treatment was approximately 40%.	BALB/c mice bearing Colon26 tumours	[90]
Chlorin-core star-shaped block copolymer (CSBC)		The combinative effects of chemotherapy and PDT -SN-38/CBSC demonstrated significant anticancer efficacy.	HT-29 xenograft model.	[91]

**Table 3:** In vitro and in vivo CRC PDT studies reported on active nanoparticle-based photosensitisers.

	In Vitro CRC PDT Studies Reported on Active Nanoparticle-Based Photosensitizers				
Nanosystem	Ligand/Moieties	PS	Key Findings	Cell Type	Ref.
EGFR-hydrogel	EGFR antibody	chlorin e6 (Ce6)	Excellent synergistic anticancer effect with increased protein expression levels.	HT-29 (Human colon cancer cell lines)	[95]
Liposomes encapsulated Ce6 and phosphoinositide 3-kinase gamma (PI3Kγ) inhibitor IPI-549	IPI-549	chlorin e6	Efficient tumour targeting, and anticancer activity	CT26 cells	[96]
Mannose-conjugated chlorin (M-chlorin)	Mannose-	M-chlorin	Higher tumour selectivity, increased cytotoxicity, and significantly suppressed tumour growth	HT29, HCT116, CT26 cells	[97]
VPA moiety-platinum diimine complexes	VPA moiety	Platinum diimine complexes	Minimal dark toxicity and improved cytotoxic effect on cancer cells	SW480 human colon cancer cell line	[98]
TPP-conjugated polymer-lipid hybrid nanoparticles	Triphenylphosphonium (TPP)	ZCNP/IR780	Enhanced specific mitochondria-targeting and enhanced anticancer effect.	Human colon carcinoma (HT-29) and HT-29 cell-bearing xenograft	[99]
	**In vivo CRC PDT studies reported on active nanoparticle-based photosensitizers**				
**Nanosystem**		**PS**	**Key Findings**	**Cell Type**	**Ref.**
Liposome encapsulated photosensitizer chlorin e6 (Ce6) and phosphoinositide 3-kinase gamma (PI3Kγ) inhibitor IPI-549	IPI-549	Ce6	The nanoformulations improved PDT therapeutic effect	CT26 cells	[96]
Verteporfin-loaded D-α-tocopheryl polyethylene glycol succinate (TPGS) nanoparticles modified with tLyp-1 tumour homing and peptide tLyp-1 decoration (t-NP)	tLyp-1 decoration (t-NP) peptide	Verteporfin (VP)	Higher tumour selectivity of PS, inhibition of tumour growth and enhanced in vivo photodynamic effects.	HCT15 colon cells	[100]

## Abbreviations

2D	Two-dimensional models
3D	Three-dimensional models
AuNPs	Gold nanoparticles
CRC	Colorectal cancer
ECM	Extracellular matrix
e6	Chlorin e6
EGFR	Epidermal growth factor receptors
FOBT	Faecal occult blood test
HpD	Hematoporphyrin derivative
mAb	Monoclonal antibodies
MCTS	Multicellular tumour spheroids
NIR	Near infrared
NPs	Nanoparticles
PDT	Photodynamic therapy
PSs	Photosensitizers
PTT	Photothermal therapy
ROS	Reactive oxygen species
TNM	Tumour, nodes, metastasis

## References

[1] Nagai H., Kim Y.H. (2017). Cancer Prevention from the Perspective of Global Cancer Burden Patterns. J. Thorac. Dis..

[2] Jiang W.G., Sanders A.J., Katoh M., Ungefroren H., Gieseler F., Prince M., Thompson S.K., Zollo M., Spano D., Dhawan P. (2015). Tissue Invasion and Metastasis: Molecular, Biological and Clinical Perspectives. Semin. Cancer Biol..

[3] Rawla P., Sunkara T., Barsouk A. (2019). Epidemiology of Colorectal Cancer: Incidence, Mortality, Survival, and Risk Factors. Prz. Gastroenterol..

[4] Fleming M., Ravula S., Tatishchev S.F., Wang H.L. (2012). Colorectal Carcinoma: Pathologic Aspects. J. Gastrointest. Oncol..

[5] Nkune N.W., Kruger C.A., Abrahamse H. (2021). Possible Enhancement of Photodynamic Therapy (PDT) Colorectal Cancer Treatment When Combined with Cannabidiol. Anticancer. Agents Med. Chem..

[6] Bevan R., Rutter M.D. (2018). Colorectal Cancer Screening—Who, How, and When?. Clin. Endosc..

[7] Simelane N.W.N., Kruger C.A., Abrahamse H. (2020). Photodynamic Diagnosis and Photodynamic Therapy of Colorectal Cancer in Vitro and in Vivo. RSC Adv..

[8] Mishra J., Dromund J., Quazi S.H., Karanki S.S., Shaw J., Chen B., Kumar N. (2013). Prospective of Colon Cancer Treatments and Scope for Combinatorial Approach to Enhanced Cancer Cell Apoptosis. Crit. Rev. Oncol. Hematol..

[9] Hong E.J., Choi D.G., Shim M.S. (2016). Targeted and Effective Photodynamic Therapy for Cancer Using Functionalized Nanomaterials. Acta Pharm. Sin. B.

[10] dos Santos A.F., de Almeida D.R.Q., Terra L.F., Baptista M.S., Labriola L. (2019). Photodynamic Therapy in Cancer Treatment-an Update Review. J. Cancer Metastasis Treat..

[11] Agostinis P., Berg K., Cengel K.A., Foster T.H., Girotti A.W., Gollnick S.O., Hahn S.M., Hamblin M.R., Juzeniene A., Kessel D. (2011). Photodynamic Therapy of Cancer: An Update. CA Cancer J. Clin..

[12] Benov L. (2014). Photodynamic Therapy: Current Status and Future Directions. Med. Princ. Pr..

[13] Hodgkinson N., Kruger C.A., Abrahamse H. (2017). Targeted Photodynamic Therapy as Potential Treatment Modality for the Eradication of Colon Cancer and Colon Cancer Stem Cells. Tumour. Biol..

[14] Kruger C., Abrahamse H. (2018). Utilisation of Targeted Nanoparticle Photosensitiser Drug Delivery Systems for the Enhancement of Photodynamic Therapy. Molecules.

[15] Kawczyk-Krupka A., Bugaj A.M., Latos W., Zaremba K., Wawrzyniec K., Kucharzewski M., Sieroń A. (2016). Photodynamic Therapy in Colorectal Cancer Treatment—The State of the Art in Preclinical Research. Photodiagn. Photodyn. Ther..

[16] Mohammad-Hadi L., MacRobert A.J., Loizidou M., Yaghini E. (2018). Photodynamic Therapy in 3D Cancer Models and the Utilisation of Nanodelivery Systems. Nanoscale.

[17] Evans C.L. (2015). Three-Dimensional in Vitro Cancer Spheroid Models for Photodynamic Therapy: Strengths and Opportunities. Front. Phys..

[18] Kruger C.A., Abrahamse H. (2019). Targeted Photodynamic Therapy as Potential Treatment Modality for the Eradication of Colon Cancer.

[19] Izci M., Maksoudian C., Manshian B.B., Soenen S.J. (2021). The Use of Alternative Strategies for Enhanced Nanoparticle Delivery to Solid Tumors. Chem. Rev..

[20] Shirasu N., Nam S.O., Kuroki M. (2013). Tumor-Targeted Photodynamic Therapy. Anticancer Res..

[21] Abrahamse H., Hamblin M.R. (2016). New Photosensitizers for Photodynamic Therapy. Biochem. J..

[22] Mesquita M.Q., Dias C.J., Gamelas S., Fardilha M., Neves M.G.P.M.S., Faustino M.A.F., Mesquita M.Q., Dias C.J., Gamelas S., Fardilha M. (2018). An Insight on the Role of Photosensitizer Nanocarriers for Photodynamic Therapy. An. Acad. Bras. Ciências.

[23] Muniyandi K., George B., Parimelazhagan T., Abrahamse H. (2020). Role of Photoactive Phytocompounds in Photodynamic Therapy of Cancer. Molecules.

[24] Lucky S.S., Soo K.C., Zhang Y. (2015). Nanoparticles in Photodynamic Therapy. Chem. Rev..

[25] Van Straten D., Mashayekhi V., de Bruijn H.S., Oliveira S., Robinson D.J. (2017). Oncologic Photodynamic Therapy: Basic Principles, Current Clinical Status and Future Directions. Cancers.

[26] De Silva P., Saad M.A., Thomsen H.C., Bano S., Ashraf S., Hasan T. (2020). Photodynamic Therapy, Priming and Optical Imaging: Potential Co-Conspirators in Treatment Design and Optimization—A Thomas Dougherty Award for Excellence in PDT Paper. J. Porphyr. Phthalocyanines.

[27] Yan J., Wang C., Jiang X., Wei Y., Wang Q., Cui K., Xu X., Wang F., Zhang L. (2021). Application of Phototherapeutic-Based Nanoparticles in Colorectal Cancer. Int. J. Biol. Sci..

[28] Castano A.P., Demidova T.N., Hamblin M.R. (2005). Mechanisms in Photodynamic Therapy: Part Two—Cellular Signaling, Cell Metabolism and Modes of Cell Death. Photodiagn. Photodyn..

[29] Panzarini E., Inguscio V., Dini L. (2011). Overview of Cell Death Mechanisms Induced by Rose Bengal Acetate-Photodynamic Therapy. Int. J. Photoenergy.

[30] Kessel D., Oleinick N.L. (2018). Cell Death Pathways Associated with Photodynamic Therapy: An Update. Photochem. Photobiol..

[31] Mahalingam S.M., Ordaz J.D., Low P.S. (2018). Targeting of a Photosensitizer to the Mitochondrion Enhances the Potency of Photodynamic Therapy. ACS Omega.

[32] Abrahamse H., Houreld N.N. (2019). Genetic Aberrations Associated with Photodynamic Therapy in Colorectal Cancer Cells. Int. J. Mol. Sci..

[33] Sekhejane P.R., Houreld N.N., Abrahamse H. (2014). Multiorganelle Localization of Metallated Phthalocyanine Photosensitizer in Colorectal Cancer Cells (DLD-1 and CaCo-2) Enhances Efficacy of Photodynamic Therapy. Int. J. Photoenergy.

[34] Song C., Xu W., Wu H., Wang X., Gong Q., Liu C., Liu J., Zhou L. (2020). Photodynamic Therapy Induces Autophagy-Mediated Cell Death in Human Colorectal Cancer Cells via Activation of the ROS/JNK Signaling Pathway. Cell Death Dis..

[35] Wei M.-F., Chen M.-W., Chen K.-C., Lou P.-J., Lin S.Y.-F., Hung S.-C., Hsiao M., Yao C.-J., Shieh M.-J. (2014). Autophagy Promotes Resistance to Photodynamic Therapy-Induced Apoptosis Selectively in Colorectal Cancer Stem-like Cells. Autophagy.

[36] Tsubone T.M., Martins W.K., Pavani C., Junqueira H.C., Itri R., Baptista M.S. (2017). Enhanced Efficiency of Cell Death by Lysosome-Specific Photodamage. Sci. Rep..

[37] Yang Y., Hu Y., Wang H. (2016). Targeting Antitumor Immune Response for Enhancing the Efficacy of Photodynamic Therapy of Cancer: Recent Advances and Future Perspectives. Oxidative Med. Cell. Longev..

[38] Anand S., Chan T.A., Hasan T., Maytin E.V. (2021). Current Prospects for Treatment of Solid Tumors via Photodynamic, Photothermal, or Ionizing Radiation Therapies Combined with Immune Checkpoint Inhibition (A Review). Pharmaceuticals.

[39] Beltrán Hernández I., Yu Y., Ossendorp F., Korbelik M., Oliveira S. (2020). Preclinical and Clinical Evidence of Immune Responses Triggered in Oncologic Photodynamic Therapy: Clinical Recommendations. J. Clin. Med..

[40] Janas K., Boniewska-Bernacka E., Dyrda G., Słota R. (2021). Porphyrin and Phthalocyanine Photosensitizers Designed for Targeted Photodynamic Therapy of Colorectal Cancer. Bioorg. Med. Chem..

[41] Sun B., Li W., Liu N. (2016). Curative Effect of the Recent Photofrin Photodynamic Adjuvant Treatment on Young Patients with Advanced Colorectal Cancer. Oncol. Lett..

[42] Kawczyk-Krupka A., Bugaj A., Latos W., Zaremba K., Wawrzyniec K., Sieron A. (2015). Photodynamic Therapy in Colorectal Cancer Treatment: The State of the Art in Clinical Trials. Photodiagn. Photodyn. Ther..

[43] Kwiatkowski S., Knap B., Przystupski D., Saczko J., Kędzierska E., Knap-Czop K., Kotlińska J., Michel O., Kotowski K., Kulbacka J. (2018). Photodynamic Therapy–Mechanisms, Photosensitizers and Combinations. Biomed. Pharmacother..

[44] Weijer R., Broekgaarden M., Kos M., van Vught R., Rauws E.A.J., Breukink E., van Gulik T.M., Storm G., Heger M. (2015). Enhancing Photodynamic Therapy of Refractory Solid Cancers: Combining Second-Generation Photosensitizers with Multi-Targeted Liposomal Delivery. J. Photochem. Photobiol. C Photochem. Rev..

[45] Baskaran R., Lee J., Yang S.-G. (2018). Clinical Development of Photodynamic Agents and Therapeutic Applications. Biomater. Res..

[46] Kou J., Dou D., Yang L. (2017). Porphyrin Photosensitizers in Photodynamic Therapy and Its Applications. Oncotarget.

[47] Zheng Y., Li Z., Chen H., Gao Y. (2020). Nanoparticle-Based Drug Delivery Systems for Controllable Photodynamic Cancer Therapy. Eur. J. Pharm. Sci..

[48] Liu R., Gao Y., Liu N., Suo Y. (2021). Nanoparticles Loading Porphyrin Sensitizers in Improvement of Photodynamic Therapy for Ovarian Cancer. Photodiagn. Photodyn. Ther..

[49] Singh S., Aggarwal A., Bhupathiraju N.V.S.D.K., Arianna G., Tiwari K., Drain C.M. (2015). Glycosylated Porphyrins, Phthalocyanines, and Other Porphyrinoids for Diagnostics and Therapeutics. Chem. Rev..

[50] Zhao L., Yang H., Amano T., Qin H., Zheng L., Takahashi A., Zhao S., Tooyama I., Murakami T., Komatsu N. (2016). Efficient Delivery of Chlorin E6 into Ovarian Cancer Cells with Octalysine Conjugated Superparamagnetic Iron Oxide Nanoparticles for Effective Photodynamic Therapy. J. Mater. Chem. B.

[51] Li Y., Yu Y., Kang L., Lu Y. (2014). Effects of Chlorin E6-Mediated Photodynamic Therapy on Human Colon Cancer SW480 Cells. Int. J. Clin. Exp. Med..

[52] Demazeau M., Gibot L., Mingotaud A.-F., Vicendo P., Roux C., Lonetti B. (2020). Rational Design of Block Copolymer Self-Assemblies in Photodynamic Therapy. Beilstein. J. Nanotechnol..

[53] Li X.-Y., Tan L.-C., Dong L.-W., Zhang W.-Q., Shen X.-X., Lu X., Zheng H., Lu Y.-G. (2020). Susceptibility and Resistance Mechanisms During Photodynamic Therapy of Melanoma. Front. Oncol..

[54] Shi R., Li C., Jiang Z., Li W., Wang A., Wei J. (2017). Preclinical Study of Antineoplastic Sinoporphyrin Sodium-PDT via In Vitro and In Vivo Models. Molecules.

[55] Laranjo M., Serra A.C., Abrantes M., Piñeiro M., Gonçalves A.C., Casalta-Lopes J., Carvalho L., Sarmento-Ribeiro A.B., Rocha-Gonsalves A., Botelho F. (2013). 2-Bromo-5-Hydroxyphenylporphyrins for Photodynamic Therapy: Photosensitization Efficiency, Subcellular Localization and in Vivo Studies. Photodiagn. Photodyn..

[56] Maduray K., Odhav B. (2012). Efficacy of Gallium Phthalocyanine as a Photosensitizing Agent in Photodynamic Therapy for the Treatment of Cancer. Proceedings of the Optics in Health Care and Biomedical Optics V.

[57] Barut B., Yalçın C.Ö., Demirbaş Ü., Özel A. (2020). Photochemical and in Vitro Phototoxic Properties of Zn (II) Phthalocyanine Bearing Piperidinium Groups on Different Cell Lines. J. Organomet. Chem..

[58] Ezquerra Riega S.D., Chiarante N., Valli F., Marino J., Roguin L.P., Awruch J., García Vior M.C. (2018). Novel Hydro- and Lipo-Philic Selenium Zinc(II) Phthalocyanines: Synthesis, Photophysical Properties and Photodynamic Effects on CT26 Colon Carcinoma Cells. Dye. Pigment..

[59] Kaleta-Richter M., Aebisher D., Jaworska D., Czuba Z., Cieślar G., Kawczyk-Krupka A. (2020). The Influence of Hypericin-Mediated Photodynamic Therapy on Interleukin-8 and -10 Secretion in Colon Cancer Cells. Integr. Cancer.

[60] Wufuer R., Ma H.-X., Luo M.-Y., Xu K.-Y., Kang L. (2021). Downregulation of Rac1/PAK1/LIMK1/Cofilin Signaling Pathway in Colon Cancer SW620 Cells Treated with Chlorin E6 Photodynamic Therapy. Photodiagn. Photodyn. Ther..

[61] Kawczyk-Krupka A., Czuba Z., Latos W., Wasilewska K., Verwanger T., Krammer B., Sieroń A. (2018). Influence of ALA-Mediated Photodynamic Therapy on Secretion of Interleukins 6, 8 and 10 by Colon Cancer Cells in Vitro. Photodiagn. Photodyn. Ther..

[62] Ma H., Yang K., Li H., Luo M., Wufuer R., Kang L. (2021). Photodynamic Effect of Chlorin E6 on Cytoskeleton Protein of Human Colon Cancer SW480 Cells. Photodiagn. Photodyn. Ther..

[63] Şueki F., Ruhi M.K., Gülsoy M. (2019). The Effect of Curcumin in Antitumor Photodynamic Therapy: In Vitro Experiments with Caco-2 and PC-3 Cancer Lines. Photodiagn. Photodyn. Ther..

[64] Costa L.D., Silva J.d.A.e., Fonseca S.M., Arranja C.T., Urbano A.M., Sobral A.J.F.N. (2016). Photophysical Characterization and in Vitro Phototoxicity Evaluation of 5,10,15,20-Tetra(Quinolin-2-Yl)Porphyrin as a Potential Sensitizer for Photodynamic Therapy. Molecules.

[65] (2021). Hypericin-Mediated Photodynamic Therapy Inhibits Growth of Colorectal Cancer Cells via Inducing S Phase Cell Cycle Arrest and Apoptosis. Eur. J. Pharmacol..

[66] Kong F., Zou H., Liu X., He J., Zheng Y., Xiong L., Miao X. (2020). MiR-7112-3p Targets PERK to Regulate the Endoplasmic Reticulum Stress Pathway and Apoptosis Induced by Photodynamic Therapy in Colorectal Cancer CX-1 Cells. Photodiagn. Photodyn. Ther..

[67] Alzeibak R., Mishchenko T.A., Shilyagina N.Y., Balalaeva I.V., Vedunova M.V., Krysko D.V. (2021). Targeting Immunogenic Cancer Cell Death by Photodynamic Therapy: Past, Present and Future. J. Immunother. Cancer.

[68] Rak J., Pouckova P., Benes J., Vetvicka D. (2019). Drug Delivery Systems for Phthalocyanines for Photodynamic Therapy. Anticancer Res..

[69] Chizenga E.P., Abrahamse H. (2020). Nanotechnology in Modern Photodynamic Therapy of Cancer: A Review of Cellular Resistance Patterns Affecting the Therapeutic Response. Pharmaceutics.

[70] dos Santos A.F., Arini G.S., de Almeida D.R.Q., Labriola L. (2021). Nanophotosensitizers for Cancer Therapy: A Promising Technology?. J. Phys. Mater..

[71] Ray P., Haideri N., Haque I., Mohammed O., Chakraborty S., Banerjee S., Quadir M., Brinker A.E. (2021). The Impact of Nanoparticles on the Immune System: A Gray Zone of Nanomedicine. J. Immunol. Sci..

[72] Montaseri H., Kruger C., Abrahamse H. (2021). Inorganic Nanoparticles Applied for Active Targeted Photodynamic Therapy of Breast Cancer. Pharmaceutics.

[73] Sadasivam M., Avci P., Gupta G.K., Lakshmanan S., Chandran R., Huang Y.-Y., Kumar R., Hamblin M.R. (2013). Self-Assembled Liposomal Nanoparticles in Photodynamic Therapy. Eur. J. Nanomed..

[74] Bakhshizadeh M., Moshirian T., Esmaily H., Rajabi O., Nassirli H., Sazgarnia A. (2017). Sonophotodynamic Therapy Mediated by Liposomal Zinc Phthalocyanine in a Colon Carcinoma Tumor Model: Role of Irradiating Arrangement. Iran. J. Basic Med. Sci..

[75] Wu R.W.K., Chu E.S.M., Huang Z., Olivo M.C., Ip D.C.W., Yow C.M.N. (2015). An in Vitro Investigation of Photodynamic Efficacy of FosPeg® on Human Colon Cancer Cells. J. Innov. Opt. Health Sci..

[76] Cheng X., Gao J., Ding Y., Lu Y., Wei Q., Cui D., Fan J., Li X., Zhu E., Lu Y. (2021). Multi-Functional Liposome: A Powerful Theranostic Nano-Platform Enhancing Photodynamic Therapy. Adv. Sci..

[77] Yao C., Zhang L., Wang J., He Y., Xin J., Wang S., Xu H., Zhang Z. (2016). Gold Nanoparticle Mediated Phototherapy for Cancer. J. Nanomater..

[78] Naidoo C., Kruger C.A., Abrahamse H. (2018). Photodynamic Therapy for Metastatic Melanoma Treatment: A Review. Technol. Cancer Res. Treat..

[79] Obaid G., Chambrier I., Cook M.J., Russell D.A. (2015). Cancer Targeting with Biomolecules: A Comparative Study of Photodynamic Therapy Efficacy Using Antibody or Lectin Conjugated Phthalocyanine-PEG Gold Nanoparticles. Photochem. Photobiol. Sci..

[80] Golombek S.K., May J.-N., Theek B., Appold L., Drude N., Kiessling F., Lammers T. (2018). Tumor Targeting via EPR: Strategies to Enhance Patient Responses. Adv. Drug Deliv. Rev..

[81] Yurt F., Sarı F.A., Ince M., Colak S.G., Er O., Soylu H.M., Kurt C.C., Avci C.B., Gunduz C., Ocakoglu K. (2018). Photodynamic Therapy and Nuclear Imaging Activities of SubPhthalocyanine Integrated TiO2 Nanoparticles. J. Photochem. Photobiol. A Chem..

[82] de Freitas C.F., Kimura E., Rubira A.F., Muniz E.C. (2020). Curcumin and Silver Nanoparticles Carried out from Polysaccharide-Based Hydrogels Improved the Photodynamic Properties of Curcumin through Metal-Enhanced Singlet Oxygen Effect. Mater. Sci. Eng. C.

[83] Ballestri M., Caruso E., Guerrini A., Ferroni C., Banfi S., Gariboldi M., Monti E., Sotgiu G., Varchi G. (2018). Core-Shell Poly-Methyl Methacrylate Nanoparticles Covalently Functionalized with a Non-Symmetric Porphyrin for Anticancer Photodynamic Therapy. J. Photochem. Photobiol. B.

[84] Bertrand N., Wu J., Xu X., Kamaly N., Farokhzad O.C. (2014). Cancer Nanotechnology: The Impact of Passive and Active Targeting in the Era of Modern Cancer Biology. Adv. Drug Deliv. Rev..

[85] Ryu J.H., Jeong Y.-I., Kim H.Y., Son G.M., Lee H.L., Chung C.-W., Chu C.W., Kang D.H. (2018). Enhanced Photosensing and Photodynamic Treatment of Colon Cancer Cells Using Methoxy Poly(Ethylene Glycol)-Conjugated Chlorin E6. J. Nanosci. Nanotechnol..

[86] Bretin L., Pinon A., Bouramtane S., Ouk C., Richard L., Perrin M.-L., Chaunavel A., Carrion C., Bregier F., Sol V. (2019). Photodynamic Therapy Activity of New Porphyrin-Xylan-Coated Silica Nanoparticles in Human Colorectal Cancer. Cancers.

[87] Chen G., Zhao Y., Xu Y., Zhu C., Liu T., Wang K. (2020). Chitosan Nanoparticles for Oral Photothermally Enhanced Photodynamic Therapy of Colon Cancer. Int. J. Pharm..

[88] Viard M., Reichard H., Shapiro B.A., Durrani F.A., Marko A.J., Watson R.M., Pandey R.K., Puri A. (2018). Design and Biological Activity of Novel Stealth Polymeric Lipid Nanoparticles for Enhanced Delivery of Hydrophobic Photodynamic Therapy Drugs. Nanomedicine.

[89] Oh I., Min H.S., Li L., Tran T.H., Lee Y., Kwon I.C., Choi K., Kim K., Huh K.M. (2013). Cancer Cell-Specific Photoactivity of Pheophorbide a-Glycol Chitosan Nanoparticles for Photodynamic Therapy in Tumor-Bearing Mice. Biomaterials.

[90] Wang S., Fan W., Kim G., Hah H.J., Lee Y.-E.K., Kopelman R., Ethirajan M., Gupta A., Goswami L.N., Pera P. (2011). Novel Methods to Incorporate Photosensitizers Into Nanocarriers for Cancer Treatment by Photodynamic Therapy. Lasers Surg. Med..

[91] Peng C.-L., Lai P.-S., Lin F.-H., Yueh-Hsiu Wu S., Shieh M.-J. (2009). Dual Chemotherapy and Photodynamic Therapy in an HT-29 Human Colon Cancer Xenograft Model Using SN-38-Loaded Chlorin-Core Star Block Copolymer Micelles. Biomaterials.

[92] Sardoiwala M.N., Kushwaha A.C., Dev A., Shrimali N., Guchhait P., Karmakar S., Choudhury S.R. (2020). Hypericin-Loaded Transferrin Nanoparticles Induce PP2A-Regulated BMI1 Degradation in Colorectal Cancer-Specific Chemo-Photodynamic Therapy. ACS Biomater. Sci. Eng..

[93] Wang K., Zhang Y., Wang J., Yuan A., Sun M., Wu J., Hu Y. (2016). Self-Assembled IR780-Loaded Transferrin Nanoparticles as an Imaging, Targeting and PDT/PTT Agent for Cancer Therapy. Sci. Rep..

[94] Gierlich P., Mata A.I., Donohoe C., Brito R.M.M., Senge M.O., Gomes-da-Silva L.C. (2020). Ligand-Targeted Delivery of Photosensitizers for Cancer Treatment. Molecules.

[95] Shanmugapriya K., Kim H., Kang H.W. (2020). Epidermal Growth Factor Receptor Conjugated Fucoidan/Alginates Loaded Hydrogel for Activating EGFR/AKT Signaling Pathways in Colon Cancer Cells during Targeted Photodynamic Therapy. Int. J. Biol. Macromol..

[96] Ding D., Zhong H., Liang R., Lan T., Zhu X., Huang S., Wang Y., Shao J., Shuai X., Wei B. (2021). Multifunctional Nanodrug Mediates Synergistic Photodynamic Therapy and MDSCs-Targeting Immunotherapy of Colon Cancer. Adv. Sci..

[97] Hayashi N., Kataoka H., Yano S., Tanaka M., Moriwaki K., Akashi H., Suzuki S., Mori Y., Kubota E., Tanida S. (2015). A Novel Photodynamic Therapy Targeting Cancer Cells and Tumor-Associated Macrophages. Mol. Cancer.

[98] Liu Z., Wang H., Zhang Z. (2021). A Valproic Acid-Modified Platinum Diimine Complex as Potential Photosensitizer for Photodynamic Therapy. J. Inorg. Biochem..

[99] Ruttala H.B., Ramasamy T., Ruttala R.R.T., Tran T.H., Jeong J.-H., Choi H.-G., Ku S.K., Yong C.S., Kim J.O. (2021). Mitochondria-Targeting Multi-Metallic ZnCuO Nanoparticles and IR780 for Efficient Photodynamic and Photothermal Cancer Treatments. J. Mater. Sci. Technol..

[100] Yang M., Jiang D., Chen Z., Chen J. (2016). Photodynamic Therapy of Drug-Resistant Human Colon Adenocarcinoma Using Verteporfin-Loaded TPGS Nanoparticles with Tumor Homing and Penetrating Peptide Functionalization. RSC Adv..

[101] Kuipers E.J., Grady W.M., Lieberman D., Seufferlein T., Sung J.J., Boelens P.G., van de Velde C.J.H., Watanabe T. (2015). Colorectal Cancer. Nat. Rev. Dis. Primers.

[102] Yuan Z., Fan G., Wu H., Liu C., Zhan Y., Qiu Y., Shou C., Gao F., Zhang J., Yin P. (2021). Photodynamic Therapy Synergize with PD-L1 Checkpoint Blockade for Immunotherapy of Colorectal Cancer by Multifunctional Nanoparticle. Mol. Ther..

[103] He C., Duan X., Guo N., Chan C., Poon C., Weichselbaum R.R., Lin W. (2016). Core-Shell Nanoscale Coordination Polymers Combine Chemotherapy and Photodynamic Therapy to Potentiate Checkpoint Blockade Cancer Immunotherapy. Nat. Commun..

[104] Xu J., Xu L., Wang C., Yang R., Zhuang Q., Han X., Dong Z., Zhu W., Peng R., Liu Z. (2017). Near-Infrared-Triggered Photodynamic Therapy with Multitasking Upconversion Nanoparticles in Combination with Checkpoint Blockade for Immunotherapy of Colorectal Cancer. ACS Nano.

[105] Seo S.-H., Kim B.-M., Joe A., Han H.-W., Chen X., Cheng Z., Jang E.-S. (2014). NIR-Light-Induced Surface-Enhanced Raman Scattering for Detection and Photothermal/Photodynamic Therapy of Cancer Cells Using Methylene Blue-Embedded Gold Nanorod@SiO2 Nanocomposites. Biomaterials.

[106] Wang X., Ouyang X., Chen J., Hu Y., Sun X., Yu Z. (2019). Nanoparticulate Photosensitizer Decorated with Hyaluronic Acid for Photodynamic/Photothermal Cancer Targeting Therapy. Nanomedicine.

[107] Edmondson R., Broglie J.J., Adcock A.F., Yang L. (2014). Three-Dimensional Cell Culture Systems and Their Applications in Drug Discovery and Cell-Based Biosensors. Assay Drug Dev. Technol..

[108] Broekgaarden M., Rizvi I., Bulin A.-L., Petrovic L., Goldschmidt R., Massodi I., Celli J.P., Hasan T. (2018). Neoadjuvant Photodynamic Therapy Augments Immediate and Prolonged Oxaliplatin Efficacy in Metastatic Pancreatic Cancer Organoids. Oncotarget.

[109] Khot M.I., Perry S.L., Maisey T., Armstrong G., Andrew H., Hughes T.A., Kapur N., Jayne D.G. (2018). Inhibiting ABCG2 Could Potentially Enhance the Efficacy of Hypericin-Mediated Photodynamic Therapy in Spheroidal Cell Models of Colorectal Cancer. Photodiagn. Photodyn. Ther..

[110] Lamberti M.J., Pansa M.F., Vera R.E., Vittar N.B.R., Rivarola V.A. (2014). Photodynamic Therapy Potentiates the Paracrine Endothelial Stimulation by Colorectal Cancer. Laser Phys..

[111] Han S.J., Kwon S., Kim K.S. (2021). Challenges of Applying Multicellular Tumor Spheroids in Preclinical Phase. Cancer Cell Int..

[112] Pereira P.M.R., Berisha N., Bhupathiraju N.V.S.D.K., Fernandes R., Tomé J.P.C., Drain C.M. (2017). Cancer Cell Spheroids Are a Better Screen for the Photodynamic Efficiency of Glycosylated Photosensitizers. PLoS ONE.

[113] Till U., Gibot L., Vicendo P., Rols M.-P., Gaucher M., Violleau F., Mingotaud A.-F. (2016). Crosslinked Polymeric Self-Assemblies as an Efficient Strategy for Photodynamic Therapy on a 3D Cell Culture. RSC Adv..

[114] Gibot L., Lemelle A., Till U., Moukarzel B., Mingotaud A.-F., Pimienta V., Saint-Aguet P., Rols M.-P., Gaucher M., Violleau F. (2014). Polymeric Micelles Encapsulating Photosensitizer: Structure/Photodynamic Therapy Efficiency Relation. Biomacromolecules.

[115] Lee J., Kim J., Jeong M., Lee H., Goh U., Kim H., Kim B., Park J.-H. (2015). Liposome-Based Engineering of Cells to Package Hydrophobic Compounds in Membrane Vesicles for Tumor Penetration. Nano Lett..

[116] Welbourn H., Duthie G., Powell J., Moghissi K. (2014). Can Photodynamic Therapy Be the Preferred Treatment Option for Anal Intraepithelial Neoplasia? Initial Results of a Pilot Study. Photodiagn. Photodyn. Ther..

[117] Zou H., Wang F., Zhou J.-J., Liu X., He Q., Wang C., Zheng Y.-W., Wen Y., Xiong L. (2020). Application of Photodynamic Therapy for Liver Malignancies. J. Gastrointest. Oncol..

[118] Vogl T.J., Eichler K., Mack M.G., Zangos S., Herzog C., Thalhammer A., Engelmann K. (2004). Interstitial Photodynamic Laser Therapy in Interventional Oncology. Eur. Radiol..

